# Parents’ Perceptions and Experiences of Parenting Programmes: A Systematic Review and Metasynthesis of the Qualitative Literature

**DOI:** 10.1007/s10567-019-00307-y

**Published:** 2019-12-10

**Authors:** J. Butler, L. Gregg, R. Calam, A. Wittkowski

**Affiliations:** 1 Division of Psychology and Mental Health, School of Health Sciences, Faculty of Biology, Medicine and Health, The University of Manchester, Manchester Academic Health Science Centre, Zochonis Building, Brunswick Street, Manchester, M13 9PL England, UK; 2 Greater Manchester Mental Health NHS Foundation Trust, Manchester, UK

**Keywords:** Metasynthesis, Thematic analysis, Acceptability, Feasibility, Parent training, Parenting, Qualitative

## Abstract

Supporting parents to meet the challenges of their caregiving role is identified as a public health concern and a priority in policies internationally. Quantitative research has established the efficacy of parenting programmes but less is understood about the key aspects that make interventions meaningful and helpful to families. We aimed to explore parents’ experiences and perceptions of parenting programmes in order to highlight the parent voice and identify key factors that parents perceive to be meaningful and improve our understanding of the acceptability and perceived benefits of parenting programmes. Six key electronic databases were searched systematically for qualitative research and eligibility for inclusion was established. A thematic synthesis was undertaken. Twenty-six studies were included, spanning 17 years of parenting research and involving 822 parents. Three main themes and nine subthemes were identified: (1) *a family’s journey* (*prior to the parenting programme, outcomes* (*including changes in the parent, child and wider family*) and *post*-*intervention*), (2) *aspects perceived to be important or valuable* (*group leader or facilitator, programme content and delivery* and *value of the group*) and (3) *challenges or difficulties* (*barriers to engagement or attendance, programme content* and *suggestions for improvement*). Reported outcomes of parenting programmes included changes in the parent alongside changes in the child and family more widely. Key recommendations to improve provision of accessible, clinically and cost-effective interventions for parents include ensuring high-quality training and supervision of facilitators, balancing flexibility and fidelity to ensure tailored content to meet individual needs, a sensitivity to parental adversity, the need for wider familial support and the availability of ongoing support following the end of a parenting programme.

## Introduction

Parenting strongly influences a child’s early life experiences and the trajectory of their cognitive, emotional, behavioural and social development across the life course (Britto et al. 2015; Leadsom et al. 2014). Supporting parents to meet the challenges of their caregiving role has consistently been identified as a public health concern; it remains a priority within international policy (Heckman 2017; Hodgkin and Newell 2007; O’Connell et al. 2009) and is considered to be a form of social investment with far-reaching social and economic implications (Balbernie 1999; Heckman 2017; Sandler et al. 2011).

Substantial evidence suggests parenting interventions, often based on social learning theory principles, have the potential to provide clinically and cost-effective methods to improve the health and well-being of parents and children (Barlow and Coren 2018; Barlow et al. 2003, 2014). A growing body of research provides evidence that parenting programmes can be effective in improving parental mental health and psychosocial functioning (Barlow et al. 2014) and improving educational (Hallam et al. 2006), emotional and behavioural outcomes amongst children (Barlow et al. 2005). The economic argument for early intervention as a means of breaking the cycle of disadvantage has also been made convincingly (Allen 2011; Bauer et al. 2014).

Research to date has largely focused on quantitative outcomes, establishing the efficacy of parenting interventions and providing a rationale for widespread implementation. However, evidence-based policy on parenting has proved difficult to implement (Law et al. 2009). A key challenge for the ‘real world’ delivery of clinically and cost-effective parenting programmes is to engage parents to participate and maximise retention (Axford et al. 2012; Bumbarger and Perkins 2008; Mytton et al. 2014). Lack of parental engagement compromises the extent to which parenting programmes are able to offer valued outcomes (Morawska and Sanders 2006). Furthermore, parents with the greatest potential to benefit may be the least likely to engage (Barrett 2010). Historically, there has been a paucity of empirical evidence examining factors relating to engagement and participation (Morawska and Sanders 2006), the successful implementation of accessible, evidence-based parenting interventions is dependent on process-orientated insights rather than just outcome data.

More recently, factors influencing parental engagement and retention has been given greater consideration (Duppong-Hurley et al. 2016; Ingoldsby 2010). Examination of the facilitators and barriers that may exist for parents has highlighted some important considerations for effective and accessible delivery of parenting programmes (Koerting et al. 2013; Miller and Prinz 2003; Mytton et al. 2014). However, previous reviews have been limited by small numbers of included studies (Koerting et al. 2013). Moreover, there is a need to go further than the examination of factors that may help and hinder parents in engaging with parenting programmes. Preliminary work has begun to consider the mechanisms by which such parenting programmes bring about improvements for parents and children (Holtrop et al. 2014). Exploring the perceptions and experiences of parents qualitatively has the potential to identify the key aspects or possible mechanisms of change that make such interventions meaningful and helpful to families (Kane et al. 2007). Qualitative analysis, which allows for the identification of the ‘critical ingredients’ that contribute to the success of parenting programmes under ‘real world’ conditions (Furlong and McGilloway 2012; Law et al. 2009), has the potential to enhance our understanding of how to adapt parenting interventions to meet parents’ needs, maximise retention and improve outcomes (Furlong and McGilloway 2012; Holtrop et al. 2014).

A systematic review of four qualitative studies by Kane et al. (2007) appears to have been the only metasynthesis of qualitative studies to date to examine parents’ experiences and perceptions of parenting programmes in order to articulate more clearly what makes these interventions meaningful to parents. This review identified key concepts: “the acquisition of knowledge, skills and understanding, together with feelings of acceptance and support from other parents in the parenting group, enabled parents to regain control and feel more able to cope. This led to a reduction in feelings of guilt and social isolation, increased empathy with the children and confidence in dealing with their behaviour” (Kane et al. 2007, p. 789). However, that review only included four studies of group-based parenting programmes in Western cultures for children with behavioural problems. As there has been a significant growth of the qualitative literature within recent years driven by the recognised value of routinely seeking the views and experiences of participants during the evaluation of parenting programmes (Mytton et al. 2014), it is timely to undertake a further and more comprehensive review of qualitative research in this area.

In line with the Medical Research Council (MRC) process evaluation framework (Moore et al. 2015), a systematic review and metasynthesis of qualitative literature would inform the development of new parenting programmes or the adaptation of existing programmes to ensure provision of parenting programmes that can meet the needs of parents and caregivers, engage and retain them in the process and enhance implementation procedures to ensure delivery is clinically and cost effective. Consequently, the current review seeks to examine what the experiences of parents and carers of parenting programmes were. Thus, the aims of the current review were to (1) provide an overview of parents’ and carers’ experiences of parenting programmes, (2) highlight the parent voice and identify key aspects of parenting programmes parents and carers perceive to be of value or not, (3) to improve our understanding of the acceptability and perceived benefits of parenting programmes.

## Methods

### Search Strategy and Identification of Studies

The SPIDER tool (Sample, Phenomenon of Interest, Design, Evaluation, Research Type) (Cooke et al. 2012) was used to develop the search strategy (see Table 1). A systematic literature search of six key electronic databases was undertaken (Applied Social Sciences Index and Abstracts (ASSIA), Medline, PsycInfo, CINHAL Plus, EMBASE and Web of Science Core Collection) from inception to the present date. Databases were searched (on 30/07/2018) for articles containing these terms in either the title, abstract or keywords. The review protocol was registered with the PROSPERO international prospective register of systematic reviews (http://www.crd.york.ac.uk/prospero, registration number CRD42018116358).

**Table 1:** Search terms and limits

1.	S—sample	(parent* OR mother* OR father* OR famil* OR carer*)
2.	PI—phenomenon of interest	(training OR intervention* OR program* OR education* OR group* OR approach*)
3.	D—design	(perce* OR perspective* OR opinion* OR experience* OR belie* OR view* OR attitude*)
4.	E—evaluation	(interview* OR focus group* OR questionnaire* OR survey*)
5.	R—research type	(qualitative OR mixed method)
6.	1 AND 2 AND 3	
7.	4 OR 5	
8.	6 AND 7	
Limits	Humans & English language	

**Fig. 1:**
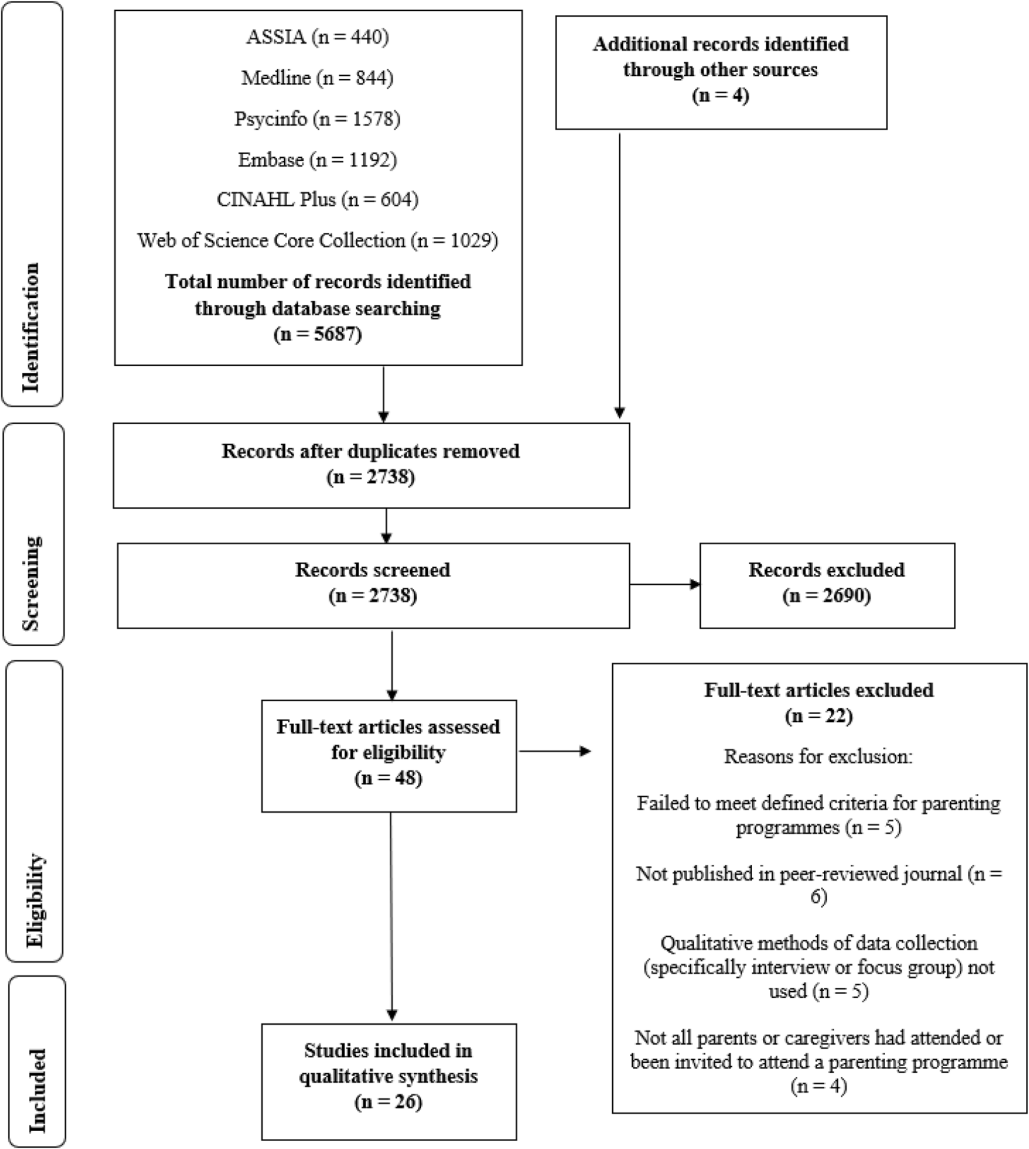
PRISMA flow diagram

Figure 1 presents an outline of the search process based on Preferred Reporting Items for Systematic Reviews and Meta-Analyses (PRISMA) guidelines (Moher et al. 2009). The initial screening of titles and abstracts was carried out by one reviewer (JB). A sample (15%) was screened by a second reviewer, independent of the research team (HA). Agreement between reviewers was 98.05%. At the full text screening stage, the first author (JB) scrutinised all papers against inclusion criteria and in the instance of uncertainty, two other authors (AW and LG) jointly scrutinised to reach agreement. Any uncertainty regarding eligibility was resolved via discussion with the research team.

### Inclusion and Exclusion Criteria

Papers were included if they (1) were written in English, (2) used qualitative methods of data collection (specifically interviews or focus groups) and analysis, (3) involved parents or caregivers who had attended or been invited to attend a parenting programme, (4) focused on parents’ views, experiences or perceptions of parenting programmes and (5) were published in a peer-reviewed journal.

Drawing upon a number of differing definitions of parenting programmes offered in the literature, the following criteria were adopted for inclusion in the current review: Interventions aimed at (1) improving parenting practices, family functioning and promoting the social and emotional well-being of children (Smith et al. 2002), (2) providing training, support or education including active skills training or coaching to parents (Mejia et al. 2012), (3) delivered in a group-setting or individually, (4) engaging parents of children aged 0–16 years. Papers were included if parents or caregivers had attended or been invited to attend a parenting programme.

Papers were excluded if the parenting programme was aimed specifically at parents of children identified as having Autism Spectrum Disorder (ASD), a learning disability or a physical disability. This decision was taken as there is substantial research evidence to suggest that the experiences and challenges faced by these parents are distinct from those parents of children without identified significant additional needs (Bourke-Taylor and Jane 2018). Whilst it is acknowledged that other populations of parents may also face unique parenting challenges, the current review sought to consider the experiences of a wide range of parents and identify possible commonalities in their experiences of parenting programmes.

### Quality Assessment

The quality of included studies was assessed by one reviewer (JB) using the 10-item Critical Appraisal Skills Programme (CASP) checklist for qualitative studies (available from https://casp-uk.net), a widely used quality assessment tool for assessing qualitative research. In order to summarise quality ratings concisely and provide a useful indicator for comparison the items on the CASP checklist were also attributed a numerical outcome (No = 0, Can’t Tell = 0.5, Yes = 1), resulting in a maximum total score of 10. The total CASP score for all papers was used to categorise the methodological quality as either ‘high’ (> 8–10), moderate (6–8) or low (≤ 5). In order to assess the reliability of quality assessment ratings 25% of the 26 included papers were rated by an independent reviewer (HA). Agreement between raters was high (95.71%, kappa = 0.87) and any disagreement was resolved via discussion.

### Thematic Synthesis

Thematic analysis, an approach often used to analyse primary qualitative data (Braun and Clarke 2006), has also been identified as an appropriate method to synthesise the findings of multiple qualitative studies (Thomas and Harden 2008). The approach was selected for use in the present review because it combines and adapts approaches from meta-ethnography (Noblit and Hare 1988) and grounded theory (Corbin and Strauss 2015; Eaves 2001), and has identified utility in allowing questions related to the appropriateness and acceptability of interventions to be addressed in order to inform policy and practice (Barnett-Page and Thomas 2009; Tong et al. 2012). The development of analytical themes allows the synthesis to ‘go beyond’ the content of the original studies and generate additional concepts or understandings (Thomas and Harden 2008; Thorne et al. 2004). The epistemological stance adopted in the current review was most closely aligned to a critical realist perspective (Fletcher 2017).

The three stages of thematic synthesis as outlined by Thomas and Harden (2008) were used: (1) Free line-by-line coding of the findings of primary studies, (2) the organisation of ‘free codes’ into related areas to construct descriptive themes and (3), finally, the development of analytical themes. All text under the headings ‘results’ or ‘findings’ were extracted electronically and entered into NVivo software (QSR International Pty Ltd. Version 12, 2018) in which data were subsequently organised ready for analysis. Comparisons were made within and across studies, meaning subsequent studies were coded into pre-existing codes and new codes were created when necessary. The process of coding and developing descriptive and analytical themes was done inductively, allowing these to emerge from the data. Guided by an experienced reviewer and clinician (AW), all stages were undertaken by the first author (JB), a white, British woman who was a trainee clinical psychologist with experience of delivering evidence-based parenting programmes. The plausibility and coherence of themes was established via review by a researcher independent to the process (RF) and via scrutiny be the research team to ensure codes and themes were appropriately derived from the data and potential bias was minimised. Guidelines enhancing the transparency in reporting the synthesis of qualitative research (ENTREQ) were adhered to (Tong et al. 2012: see Table 4 for completed checklist).

## Results

### Characteristics of Included Studies

A total of 26 studies was identified for inclusion in the current review as summarised in Table 2. Despite no time limit being applied to the search, included studies were all conducted in or after between 2001. They considered a variety of parenting programmes, the most frequently cited being (1) the Triple P Positive Parenting Programme, including groups, seminars and amended versions of Triple P (n = 7) (Coates et al. 2017; Cullen et al. 2013; Errázuriz et al. 2016; Garcia et al. 2018; Haskett et al. 2018; Houlding et al. 2012; Lewis et al. 2016), (2) Webster-Stratton’s Incredible Years Parent Training Program (*n* = 6) (and 3) Strengthening Families Program 10–14 (*n* = 3) Twenty-five of the included studies referred to parenting programmes delivered in a group format with only one being delivered individually. Studies were conducted in the United States (*n* = 10), the United Kingdom (*n* = 8), Canada (*n* = 2), Panama (*n* = 2), Ireland (*n* = 2), Australia (*n* = 1) and Chile (*n *= 1).

**Table 2:** Characteristics of included studies

	Authors, publication year, country	Aims/objectives/research questions	Participants^a^	Intervention	Data collection^b^	Method of analysis	Main themes identified^c^
1	Wilson et al. (2018), UK	(1) Examine the parenting and help-seeking experiences of parents affected by personality disorder, (2) explore the acceptability of Helping Families Programme to this population, (3) refine the protocol for the subsequent pilot RCT	*N *= 5 mothers (who met diagnostic criteria for personality disorder and their children met criteria for a behavioural and/or emotional problem)	Helping Families Programme (Day et al. 2011)	Semi-structured interviews	Interpretive phenomenological analysis (Smith and Osborn 2008)	(1) The experience of parenthood, (2) Being a parent affected by personality disorder, (3) Experience of the intervention, (4) Qualities of helping
2	Garcia et al. (2018), USA	(1) What inner and outer contextual factors influence access to and active engagement in Triple P?, 2) To what extent do they believe Triple P is effective in addressing children’s maladaptive behaviours and promoting positive parent–child interactions?	*N *= 35 parents (aged 20–49 years) referred to child-welfare agencies	Group Triple P (https://www.triplep.net)	Interview & Focus Groups	Grounded theory (Strauss and Corbin 1990)	(1) Barriers to engagement, (2) Overcoming barriers to engagement, (3) Effects of engagement: New insights and actions about effective parenting
3	Haskett et al. (2018), USA	To examine the degree to which parents experiencing homelessness considered Triple P content, materials and delivery methods to be relevant and helpful	*N *= 16 parents experiencing homelessness	Triple P Seminar (https://www.triplep.net)	Focus groups	Content analysis (Flick 2014)	(1) Relevance of the Triple P seminar to the parenting experience in shelters, (2) Parenting reflections and challenges, (3) Parents’ opinions about the seminar format and materials, (4) Parents recommended changes to the seminar
4	Coates et al. (2017), Australia	To gain the perspectives of parents who have completed the program	*N *= 18 parents self-identifying as having a mental health difficulty	Mental Health Positive Parenting Program (MHPPP) (Phelan et al. 2013)	Semi-structured telephone interviews	Thematic analysis (Braun and Clarke 2006)	(1) Being in a group with others with mental illness, (2) Focus on child development and parenting with a mental illness, (3) The home visits
5	Hartwig et al. (2017)*, USA	To allow participants to describe their experience with the programme in their own words and examine how participating mothers described parenting following involvement in the program	*N *= 166 low-income mothers, predominantly Hispanic and Black	Legacy for Children (Kaminski et al. 2013)	Focus Groups	Grounded theory (Hennink et al. 2011)	(1) Commitment to parenting, (2) Nurturance & sensitivity/responsivity, (3) Parental control, (4) Developmental stimulation
6	Errázuriz et al. (2016), Chile	Evaluate the feasibility of implementing Triple P in Chile, and to assess its social and cultural acceptability, the level of involvement of families, the costs involved, and the impact of children and their families	*N *= 34 parent attending primary care centres in Santiago de Chile	Group Triple P (https://www.triplep.net)	Focus groups	Grounded theory (Strauss and Corbin 2002)	(1) Impact of the program: re-learning how to parent, relationship with children, family dynamics, changes in children, (2) Program implementation: materials and activities, home exercises, child caretakers, suggestions
7	Duppong-Hurley et al. (2016), USA	(1) To learn about barriers to participation faced by families who had enrolled in, but never or minimally attended, a community- based parenting program, (2) Gather feedback from these parents who did not participate in the small-group parenting class regarding their perspective about alternative, web-based methods of delivery	*N *= 27 parents who signed up for but did not complete a community-based parenting program	Common-Sense Parenting (https://www.boystown.org/parenting/Pages/common-sense-parenting.aspx)	Semi-structured telephone interviews	Not specified	(1) Reasons for registering for the parent program, (2) Barriers to attending the parenting program, (3) What would have helped the parents attend the program
8	Lewis et al. (2016), USA	To explore child-welfare involved parents’ perceptions of the relevance and fit of Pathways Triple P, to their needs	*N* = 47 parents involved with the state child-welfare agency	Pathways Triple P (https://www.triplep.net)	Semi-structured interviews	Thematic Analysis (framework method) (Ritchie and Lewis 2003)	(1) Program content, (2) Program materials, (3) Program structure, (4) Endorsements, (5) Barriers to participation
9	Mejia et al. (2016), Panama	To explore parental perceptions of cultural fit	*N* = 30 Panamanian parents of adolescents	Strengthening families programme (SFP) 10-14 (Molgaard and Spoth 2001)	Semi-structured interviews	Thematic Analysis (Braun and Clarke 2006)	(1) Communication, (2) Resilience, (3) Community specific concerns, (4) Cross-cultural concerns
10	Vella et al. (2015), UK	Examine in depth the experiences and reflective views of parents who have attended a ‘Understanding Your Child’s Behaviour’ (UYCB) group to understand how parents made sense of participating in the group, whether they have been able to implement new knowledge and skills and participation may have been relevant approximately 10 months after completion	*N *= 10 parents aged 18+	Solihull Approach parenting group: ‘Understanding Your Child’s Behaviour’ (UYCB) (https://solihullapproachparenting.com/)	Semi-structured interviews	Interpretive Phenomenological Analysis (Smith et al. 2009)	(1) Two tiers of satisfaction, (2) Development as a parent, (3) Improved self-belief, (4) Follow-up: the ‘Matthew Effect’
11	Furlong and McGilloway (2015)*, Ireland	To assess longer term experiences of the Incredible Years BASIC Preschool/Early School Years Parent Training Programme (IYPP) within socially deprived settings in Ireland, with a key focus on investigating the key facilitative and inhibitive factors associated with trial outcomes	*N* = 28 Caucasian Irish parents	Incredible Years BASIC Preschool/Early School Years Parent Training Programme (IYPP) (http://www.incredibleyears.com/)	Semi-structured interviews	Constructivist Grounded Theory (Charmaz 2006)	(1) Maintaining positive outcomes, (2) Relapse in positive outcomes, (3) Diverging paths
12	Mejia et al. (2015), Panama	To explore parents’ perceptions and beliefs about changes after taking part in the program	*N* = 30 Panamanian parents of adolescents	Strengthening Families Programme (SFP) 10-14 (Molgaard and Spoth 2001)	Semi-structured interviews	Thematic Analysis (Braun and Clarke 2006)	(1) Changes in the child, (2) Changes in the Parent, (3) Changes in the couple, (4) Changes in the interaction
13	Butcher and Gersch (2014), UK	To understand the qualitative experiences of parents of children in the early years who were identified as being socially isolated and/or having difficulties relating to their child and had taken part in the Time Together home visiting intervention	*N* = 7 white British parents aged 26–35 years	Time Together [drawing upon principles of Peers Early Education Partnership (Evangelou and Sylva 2011) and the Solihull approach (Douglas and Ginty 2001)]	Semi-structured interviews	Interpretive phenomenological Analysis (Smith and Osborn 2008)	(1) The notion of self, (2) The power of play, (3) Influential relationships, (4) The meaning of social isolation
14	Holtrop et al. (2014), USA	To provide a better understanding of the process of change within an evidence-based parent training intervention: What is the process through which parents’ experiences in the PMTO intervention led to change in their parenting practices?	N = 20 white parents aged 28–64 years	Parent Management Training—the Oregon Model (PMTO™) (Forgatch and Patterson 2008)	Semi-structured interviews	Grounded Theory (Glaser and Strauss 1967)	(1) PMTO process of change, (2) Content of PMTO, (3) PMTO method of delivery, (4) Additional characteristics
15	Estefan et al. (2013), USA	To explore the nature and co-occurrence of family stressors in a sample of parents involved in the child-welfare system who have been referred to an intensive therapeutic parent training program	*N* = 21 parents involved or at risk of becoming involved in the child-welfare system	Nurturing Parents Program (NPP) (https://www.nurturingparenting.com/)	Semi-structured interviews	Not specified	(1) Change methods of discipline, (2) Better understanding/tools for coping with anger, (3) Challenges with implementing new parenting practices, (4) Learnt new skills, (5) Felt well supported by facilitators and group format
16	Cullen et al. (2013), UK	To examine the important factors with respect to intervention and the experiences of both parents and those involved in the delivery of the programmes	*N* = 133 parents participating in parenting programmes across local authorities in England	Triple P; Incredible Years; Families and Schools Together (FAST); and the Strengthening Families Programme 10-14 (https://www.triplep.net; http://www.incredibleyears.com/; https://www.familiesandschools.org/; Molgaard and Spoth 2001)	Semi-structured interviews	Thematic Analysis (reference not provided)	(1) Family issues, (2) Parenting courses as educational processes
17	Houlding et al. (2012), Canada	To examine the perceived impact, cultural acceptability and experience of the Group Triple P Positive Parenting Program	*N *= 11 Aboriginal Canadian parents	Group Triple P Parenting Programme (https://www.triplep.net)	Semi-structured interviews	Interpretive Phenomenological Analysis (Colaizzi 1978)	(1) The helpfulness of the program, (2) How parenting behaviours changed, (3) How children’s behaviour changed, (4) Processes that facilitated learning, (5) Benefits of the group format, (6) Cultural acceptability of strategies and process, (7) Cultural acceptability of indigenous resources
18	Furlong and McGilloway (2012)*, Ireland	To explore: (1) which aspects of the program were most valued by parents and perceived as producing positive changes; (2) what challenges they encountered in learning the new skills and (3) the experiences of the small number of parents who dropped out of the program	*N* = 33 parents (31 mothers and 2 fathers with a mean age 34 years)	Incredible Years BASIC Preschool/Early School Years Parent Training Program (IYP) (http://www.incredibleyears.com/)	Semi-structured interviews	Constructivist Grounded Theory (Charmaz 2006)	(1) Mechanisms of change, (2) Trials of parenting, (3) ‘Failure to launch’
19	Bermudez et al. (2011), USA	To gain an in-depth understanding of the experiences of Mexican–American mother’s participation in a parent education programme	*N* = 20 Mexican–American mothers parenting alone	Parenting Through Change (PTC) (Forgatch and DeGarmo 1999)	Semi-structured interviews	Heuristic Inquiry (Moustakas 1990)	(1) Participants gained valuable knowledge related to child rearing practices, (2) Participants gained a heightened awareness about themselves as mothers, (3) Class process was important, (4) Experiences of taking the classes varied for sample, (5) Interview process was meaningful and empowering, (6) The researchers’ experiences were meaningful and empowering
20	Owens et al. (2007), USA	To examine parents’ perceptions of barriers to participation, strengths and weaknesses of the program and recommendations for future programming	*N* = 15 Caucasian parents	Community-based, behavioural parenting program (derived from Defiant Child program (Barkley 1997) and the Community-Orientated Parenting Education (COPE) Programme (Cunningham et al. 1996)	Focus groups	Focus Group Toolkit (Morgan and Krueger 1998)	1) Strengths of the parenting program, (2) Weaknesses of the parenting program, (3) Barriers to participation in parenting groups, (4) Recommendations for improvement of the program
21	Russell et al. (2007), Canada	To determine parent views regarding the beneficial and detrimental aspects of the multi-faceted interventions they received	N = 24 parents culturally diverse parents who were referred by child protection agencies	Project Parent (no reference provided)	Focus groups	Grounded Theory (Strauss and Corbin 1990)	Major theme: reciprocal multi-system interventions, (1) Parent psychological level: affirming parent self-worth, (2) Parent–child level: non-directive instruction, (3) Social-family level: promoting social connections, (4) Social system level: empowering communication
22	Patterson et al. (2005)*, UK	To report the usefulness of the programme to the parents, aspects they found helpful and why, and the extent to which they had observed changes in their own and their children’s mental health and behaviour as a result of the programme	*N* = 26 parents (22 who attended at least 50% of the programme, 3 non-attenders and 1 who ‘dropped out’)	Webster-Stratton ‘Parents and Children Series’ programme (http://www.incredibleyears.com/)	Semi-structured interviews	Grounded theory (Glaser and Strauss 1967)	(1) Parents needs and problems, (2) Ways in which the programme had an impact on these needs and problems, (3) Programme delivery, (4) Aspects of the programme with which some parents disagreed, (5) Needs not met by the programme.
23	Mockford and Barlow (2004), United Kingdom	To look at the effect of a parenting programme on everyday family lives. In particular, the effects the parenting programme may have on both parents when only one parent, mostly the mother, attends the programme	*N* = 14 mothers	Webster-Stratton ‘Parents and Children Series’ programme (http://www.incredibleyears.com/)	Semi-structured interviews	Constant Comparative Method (Glaser and Strauss 1967)	(1) Difficulties in ‘engaging the partner’ and reluctance to attend the programme, (2) Difficulties in changing the established habits of their partners, (3) Findings the time to parent together
24	Stewart-Brown et al. (2004)*, UK	To test the effectiveness at one year of the Webster-Stratton Parents and Children Series group parenting programme in a population sample of parents	*N* = 26 intervention group parents	Parent and Child Series Incredible Years programme (http://www.incredibleyears.com/)	Semi-structured interviews	Grounded Theory (Glaser and Strauss 1967)	(1) Impact on intervention group parents, (2) Specific improvements in their children’s behaviour, (3) Improvement in their relationship with their child, (4) Difficulties with the programme.
25	Wolfe and Haddy (2001), USA	To inform and improve parent education efforts by providing insight about participants’ perceived experiences and programme impact	*N* = 15 mothers (11 White women and 4 African American Women)	Listening to Children (LTC) (Wolfe 1999)	Semi-structured interviews	Content Analysis (reference not provided)	(1) Increased social support, (2) Heightened self-awareness, (3) Improved parenting skills, (4) Enhanced sense of empowerment.
26	Barlow and Stewart-Brown (2001)*^d^, UK	To gain a better understanding of parents’ experiences of a parenting programme (e.g. whether parents had found taking part in a group with other parents helpful and, if so, in what ways).	*N* = 11 parents who had attended at least 90% of the programme	Family Links Nurturing Programme (https://familylinks.org.uk/the-nurturing-programme)	Semi-structured interviews	Not specified	(1) Reasons for participating in the programme, (2) Overall feelings and thoughts about the programme, (3) Ways in which parents benefited from taking part in a parenting programme, (4) Support in the role of a parent, (5) Regaining feelings of control, (6) Increased feelings of empathy and ability to identify with their children, (7) Aspects of the programme that parents did not like.

^a^Only data from participants that meet the inclusion criteria for the review are presented

^b^Other methods of data collection may have been used in the included studies but only data gathered from interviews or focus groups is included in the review

^c^Only themes derived from parent interviews or focus groups have been included

^d^In studies marked with an * qualitative data had been collected as part of a larger funded randomised control trial

Whilst a number of the included studies employed a range of methods of data collection, qualitative data were derived from interviews (*n *= 20) or focus groups (*n *= 6). In six of the included studies it was possible to identify that qualitative data had been collected as part of a larger randomised control trial. The most common methods of analysis were Grounded Theory (*n *= 9), Thematic Analysis (*n *= 5) and Interpretive Phenomenological Analysis (*n *= 4). A number of studies (*n *= 3) did not specify the method of analysis used but described the analytical process used. The sample sizes of the 26 included studies were diverse, ranging from *n* = 5 (Wilson et al. 2018) to *n* = 166 (Hartwig et al. 2017). The review includes data from a total of 822 parents. Interventions included in the review were offered to a variety of parents including specific sub-groups (e.g. parents experiencing mental health difficulties, homelessness, parents involved in child-welfare agencies, lone parents and low-income parents).

### Methodological Quality of Included Studies

Overall, the methodological quality of all included studies was deemed either high (*n *= 22) or moderately high (*n *= 4) (see Table 3 for details). However, there were a number of issues that were identified. There were only six studies (23%) in which the relationship between researcher and participant had been adequately considered and reported. In eleven (42%) of the included studies approval by an ethics committee was not evidenced and in four (15%) of these, there was no evidence that ethical issues had been taken into consideration.

**Table 3:** Methodological quality assessment of included studies

	Authors and publication year	1. Was there a clear statement of the aims of the research?	2. Is a qualitative methodology appropriate?	3. Was the research design appropriate to address the aims of the research?	4. Was the recruitment strategy appropriate to the aims of the research?	5. Was the data collected in a way that addressed the research issue?	6. Has the relationship between researcher and participants been adequately considered?	7. Have ethical issues been taken into consideration?	8. Was the data analysis sufficiently rigorous?	9. Is there a clear statement of findings?	10. How valuable is the research?	Total score (max score = 10)
1	Wilson et al. (2018)	1 (Yes)	1 (Yes)	1 (Yes)	1 (Yes)	1 (Yes)	1 (Yes)	1 (Yes)	1 (Yes)	1 (Yes)	1 (Yes)	10 (High)
2	Garcia et al. (2018)	1 (Yes)	1 (Yes)	1 (Yes)	1 (Yes)	1 (Yes)	1 (Yes)	0.5 (Can’t^a^ Tell)	1 (Yes)	1 (Yes)	1 (Yes)	9.5 (High)
3	Haskett et al. (2018)	1 (Yes)	1 (Yes)	1 (Yes)	1 (Yes)	1 (Yes)	0.5 (Can’t Tell)	1 (Yes)	1 (Yes)	1 (Yes)	1 (Yes)	9.5 (High)
4	Coates et al. (2017)	1 (Yes)	1 (Yes)	1 (Yes)	1 (Yes)	1 (Yes)	1 (Yes)	1 (Yes)	1 (Yes)	1 (Yes)	1 (Yes)	10 (High)
5	Hartwig et al. (2017)	1 (Yes)	1 (Yes)	1 (Yes)	1 (Yes)	1 (Yes)	0.5(Can’t Tell)	1 (Yes)	1 (Yes)	1 (Yes)	1 (Yes)	9.5 (High)
6	Errazuriz et al. (2016)	1 (Yes)	1 (Yes)	1 (Yes)	1 (Yes)	1 (Yes)	0.5 (Can’t Tell)	1 (Yes)	1 (Yes)	1 (Yes)	1 (Yes)	9.5 (High)
7	Duppong-Hurley et al. (2016)	1 (Yes)	1 (Yes)	1 (Yes)	1 (Yes)	1 (Yes)	0 (No)	0.5 (Can’t Tell)	0.5 (Can’t Tell)	1 (Yes)	1 (Yes)	8 (High)
8	Lewis et al. (2016)	1 (Yes)	1 (Yes)	1 (Yes)	1 (Yes)	1 (Yes)	0 (No)	1 (Yes)	1 (Yes)	1 (Yes)	1 (Yes)	9 (High)
9	Mejia et al. (2016)	1 (Yes)	1 (Yes)	1 (Yes)	1 (Yes)	1 (Yes)	0.5 (Can’t Tell)	0.5 (Can’t Tell)	1 (Yes)	1 (Yes)	1 (Yes)	9 (High)
10	Vella et al. (2015)	1 (Yes)	1 (Yes)	1 (Yes)	1 (Yes)	1 (Yes)	0.5 (Can’t Tell)	1 (Yes)	1 (Yes)	1 (Yes)	1 (Yes)	9.5 (High)
11	Furlong and McGilloway (2015)	1 (Yes)	1 (Yes)	1 (Yes)	1 (Yes)	1 (Yes)	0.5 (Can’t Tell)	1 (Yes)	1 (Yes)	1 (Yes)	1 (Yes)	9.5 (High)
12	Mejia et al. (2015)	1 (Yes)	1 (Yes)	1 (Yes)	1 (Yes)	1 (Yes)	0.5 (Can’t Tell)	0.5 (Can’t Tell)	1 (Yes)	1 (Yes)	1 (Yes)	9 (High)
13	Butcher and Gersch (2014)	1 (Yes)	1 (Yes)	1 (Yes)	1 (Yes)	1 (Yes)	1 (Yes)	1 (Yes)	1 (Yes)	1 (Yes)	1 (Yes)	10 (High)
14	Holtrop et al. (2014)	1 (Yes)	1 (Yes)	1 (Yes)	1 (Yes)	1 (Yes)	1 (Yes)	1 (Yes)	1 (Yes)	1 (Yes)	1 (Yes)	10 (High)
15	Estefan et al. (2013)	0.5 (Can’t Tell)	0.5 (Can’t Tell)	0.5 (Can’t Tell)	0.5 (Can’t Tell)	0.5 (Can’t Tell)	0 (No)	1 (Yes)	1 (Yes)	1 (Yes)	1 (Yes)	6.5 (Moderate)
16	Cullen et al. (2013)	1 (Yes)	1 (Yes)	1 (Yes)	0.5 (Can’t Tell)	0.5 (Can’t Tell)	0 (No)	1 (Yes)	0.5 (Can’t Tell)	1 (Yes)	1 (Yes)	7.5(Moderate)
17	Houlding et al. (2012)	1 (Yes)	1 (Yes)	1 (Yes)	1 (Yes)	1 (Yes)	1 (Yes)	1 (Yes)	1 (Yes)	1 (Yes)	1 (Yes)	10 (High)
18	Furlong and McGilloway (2012)	1 (Yes)	1 (Yes)	1 (Yes)	1 (Yes)	1 (Yes)	0.5(Can’t Tell)	0.5 (Can’t Tell)	1 (Yes)	1 (Yes)	1 (Yes)	9 (High)
19	Bermudez et al. (2011)	1 (Yes)	1 (Yes)	1 (Yes)	1 (Yes)	1 (Yes)	0.5 (Can’t Tell)	0 (No)	1 (Yes)	1 (Yes)	1 (Yes)	8.5 (High)
20	Owens et al. (2007)	1 (Yes)	1 (Yes)	1 (Yes)	1 (Yes)	1 (Yes)	0.5 (Can’t Tell)	0.5 (Can’t Tell)	1 (Yes)	1 (Yes)	1 (Yes)	9 (High)
21	Russell et al. (2007)	1 (Yes)	1 (Yes)	1 (Yes)	1 (Yes)	1 (Yes)	0 (No)	0 (No)	0.5 (Can’t Tell)	1 (Yes)	1 (Yes)	7.5 (Moderate)
22	Patterson et al. (2005)	1 (Yes)	1 (Yes)	1 (Yes)	1 (Yes)	0.5 (Can’t Tell)	0.5 (Can’t Tell)	1 (Yes)	1 (Yes)	1 (Yes)	1 (Yes)	9 (High)
23	Mockford and Barlow (2004)	1 (Yes)	1 (Yes)	1 (Yes)	1 (Yes)	1 (Yes)	0 (No)	0.5 (Can’t Tell)	0.5 (Can’t Tell)	1 (Yes)	1 (Yes)	8 (High)
24	Stewart-Brown et al. (2004)	1 (Yes)	0.5 (Can’t Tell)	1 (Yes)	1 (Yes)	1 (Yes)	0 (No)	1 (Yes)	0.5 (Can’t Tell)	1 (Yes)	1 (Yes)	8 (High)
25	Wolfe and Haddy (2001 (Yes))	1 (Yes)	1 (Yes)	1 (Yes)	1 (Yes)	1 (Yes)	0 (No)	0 (No)	0.5 (Can’t Tell)	1 (Yes)	1 (Yes)	7.5 (Moderate)
26	Barlow and Stewart-Brown (2001)	1 (Yes)	1 (Yes)	1 (Yes)	1 (Yes)	1 (Yes)	0.5 (Can’t Tell)	0 (No)	0.5 (Can’t Tell)	1 (Yes)	1 (Yes)	8 (High)
	% of Included studies rated as 1:‘Yes’	96	92	96	92	88	23	58	73	100	100	

^a^‘Can’t tell’ indicates the required information was unclear or evidence provided by study authors in the text was insufficient

Given that there is not a widely accepted or empirically tested approach for excluding qualitative studies from synthesis on the basis of quality (Dixon-Woods et al. 2006; Thomas and Harden 2008), no studies were excluded.

### Thematic Synthesis

**Fig. 2:**
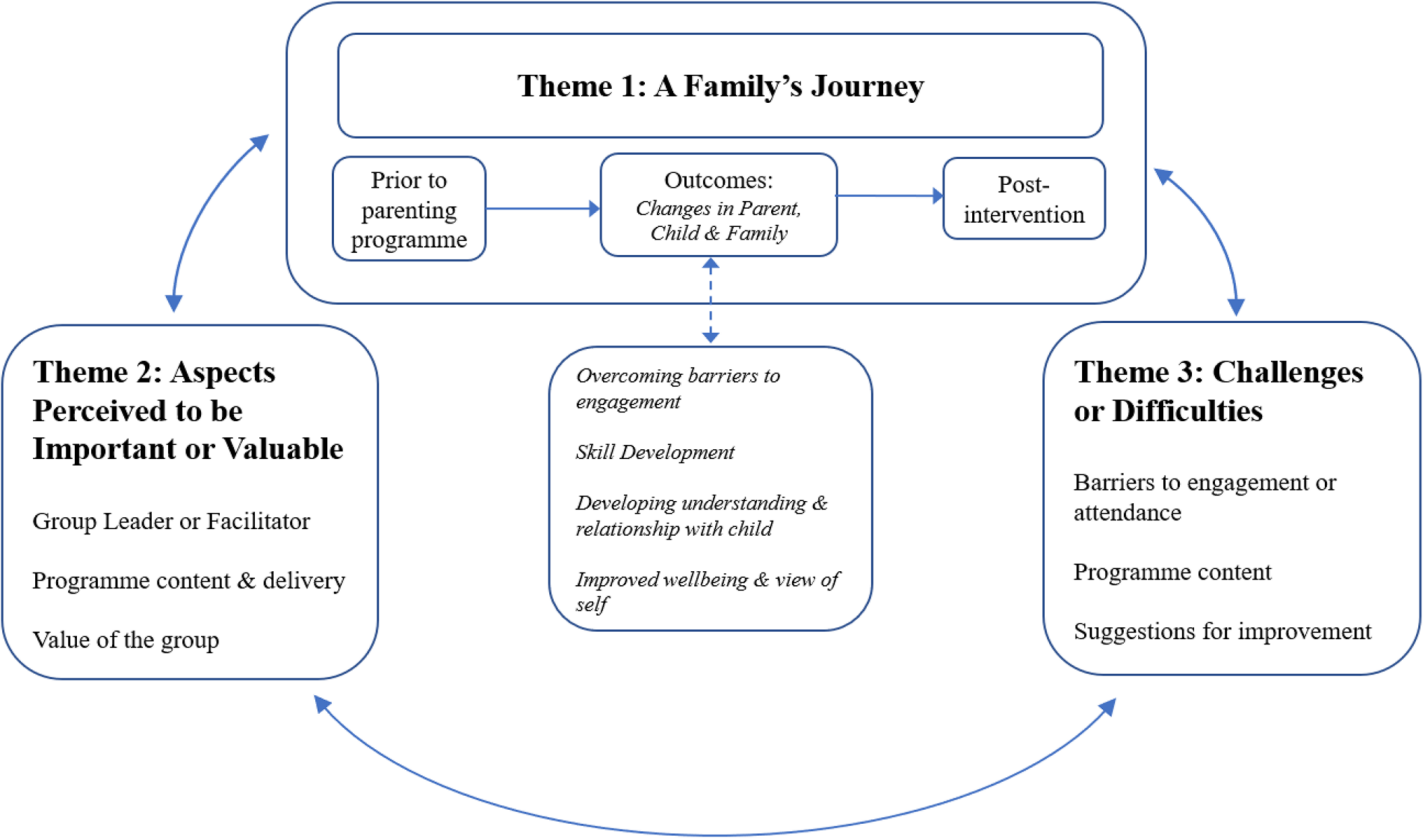
Diagram depicting themes and subthemes in the thematic synthesis

Three main themes were developed during the synthesis representing different aspects of parents’ perceptions and experiences of parenting programmes: (1) *a family’s journey,* (2) *aspects perceived to be important or valuable* and (3) *challenges or difficulties*. A *family’s journey* included subthemes relating to perceptions and experiences prior to the parenting programme; outcomes associated with the parenting programme and post-intervention experiences. Outcomes included changes in the parent (including overcoming barriers to engagement, skill development, developing understanding and relationship with the child, improved well-being and view of self), alongside changes in the child and family more widely. *Aspects of the parenting programmes perceived to be important or valuable* included factors related to the group leader or facilitator, programme content and delivery and the group. Subthemes included within *challenges and difficulties* associated with the parenting programme included barriers to engagement or attendance, programme content and suggestions parents made for improving the programme. A detailed matrix of themes is presented in Table 5, illustrating which themes were present in the 26 included studies. The themes and their relation to one another are depicted in Fig. 2. A family’s journey through a parenting programme is influenced by their experience of the aspects perceived to be important or valuable and the challenges and difficulties they face in engaging in such programmes. Moreover, it is hypothesised that the outcomes associated with changes in the parent have a reciprocal relationship with changes in child and family more widely.

### Theme 1: A Family’s Journey

#### Subtheme 1.1: Prior to the Parenting Programme

This subtheme related to the experiences and perceptions of parents prior to commencing a parenting programme. Parents described experiencing a range of difficulties including problems managing their child’s behaviour, problems in the relationship with their child, frequent distressing interactions with their child and feeling isolated. Parents commonly described a sense of helplessness, desperation and feeling overwhelmed or ‘out of control’. One parent noted, “we were all overwhelmed, really, we all arrived here very desperate” (Errázuriz et al. 2016, p. 3444) and another “I failed completely to find a way to cope… I felt like I was out of control” (Patterson et al. 2005, p. 57).

A number of authors noted that parents feared being judged to be a ‘bad parent’ and felt obligated to participate in a parenting programme, acquiescing despite initial pessimism about how effective it would be. Such feelings were more common amongst parents that were mandated to attend as part of child-welfare processes. In contrast, other parents saw the invitation to attend a parenting programme as a recognition of the difficulties they were experiencing. A frequently cited reason for attending a parenting programme was a commitment to “be a better parent” (Hartwig et al. 2017, p. 506).

#### Subtheme 1.2: Outcomes

##### Subtheme 1.2.1: Changes in Parent

Subtheme 1.2.1.1: Overcoming Barriers to Engagement: Parents commonly described a shift from the initial pessimism or reluctance, described prior to the parenting programme, to an intrinsic willingness to participate:

“So like I said, the first two sessions I’m like whatever, I gotta come here. I don’t feel like being here, but after the third or fourth session it really made me want to be here more ‘cause I wanted to learn and figure out what did I do wrong or what was I not doing right with these kids.” (Garcia et al. 2018, p. 292)

“At first, like the first couple of weeks, I was like I can’t believe I have to do this, and it’s ridiculous. But it was all right. I mean the group, we got to know the people in our group and stuff, and they were people just like us. There was a couple that was our age, couples that were older. I liked the group thing, the way it was set up like that.” (Estefan et al. 2013, p. 206)

Subtheme 1.2.1.2: Skill Development: Parents frequently described acquisition of new skills and the reinforcement of existing skills as an outcome of attending the parenting programme. Some of the key skills that parents reported were learning emotional regulation strategies to support them to remain calm resulting in a reduction in shouting, physical punishment and the use of punitive parenting strategies. In turn, parents were able to employ the alternative strategies they learnt on the parenting programme:

We learned about escalating, that it is not necessary to yell and keep punishing, that you need to make clear, precise rules and for the child to understand you so that things work. (Errázuriz et al. 2016, p. 3445)

I do a lot less shouting and I’d occasionally smack but I don’t do that now… learning to reward rather than punish, I don’t think we hardly [ever] punish now, do we… (Barlow and Stewart-Brown 2001, p. 124)

Subtheme 1.2.1.3: Developing Understanding and the Relationship with Child: Parents commonly reported an improved relationship with their child as an outcome of attending a parenting programme. In addition, parents described improved communication with their child, increased capacity to empathise with their child, feeling closer to their child, increased affection, prioritising spending time playing with their child, establishing age-appropriate expectations, recognising the importance of listening to their child and seeking an understanding of their behaviour:

It’s just completely changed both of us, I think, in our outlook to each other as well. We’re enjoying each other’s company now. We’re not just arguing constantly. It’s changed our lives. It really has given me my daughter back. (Cullen et al. 2013, p. 1037)

Um, the one thing that I would say was the most helpful was that I recognized that my children have the same feelings and anxieties as adults have, and for some reason I think adults have this misconception that they can speak to children any way that they like. That they don’t have the [same] feelings, you know, and I think that has been really helpful for me, just to recognize that sometimes [that] they need to talk about things as well. And it is often harder for kids to talk about things because they don’t have the vocabulary, they don’t have the words to express the way that they are feeling, and that it is up to me to try and [help them to] express how they are feeling, you know. And I think that more than anything else has been a benefit. (Barlow and Stewart-Brown 2001, p. 125)

Subtheme 1.2.1.4: Improved Well-being and View of Self: Parents described feeling empowered, gaining confidence in their parenting ability and a sense of regaining control. Moreover, parents reported a heightened self-awareness, reduction in self-criticism, feelings of guilt and recognising a need for self-care:

I think my biggest hurdle has been looking at my kids and being able to say I’m okay. I do good things for you. I may not be perfect—but I am okay. I think that for me that was the biggest hurdle—just to get over the fact that I am not horrible. (Russell et al. 2007, p. 108)

I just felt as well that it made me recognize that I was a human being as well, you know. And I have needs and requirements as well, [] whereas before I was trying to be the super-duper wonderful parent, trying to do everything without actually paying any attention to myself. I think I recognized that, yes, I can still be a good parent but still look after myself as well. So I think recognizing that was good for me. (Barlow and Stewart-Brown 2001, p. 123)

Central to this process for many parents was reflecting on their own experiences of being parented, recognising this influence on their approach to parenting and the challenge of breaking this intergenerational cycle. Parents identified how difficult and distressing this process can be:

It was hard initially because I was forced to look at things at happened when I was raised. I had to resolve some of my own issues and that’s hard for people to do. So I was able to learn to get over my own childhood, so I’m not reliving my own childhood through my kids. It’s hard to break the cycle and do something different, but we’re for the most part doing it. (Wolfe and Haddy 2001, p. 82)

I think I would have given up the course if I hadn’t had the counsellor because it was too much at one point … I was jealous of the kids … And I think a lot of parents there haven’t had the perfect upbringing and I think there’s certain things that could come up out of the course that could upset a lot of people. (Furlong and McGilloway 2012, p. 623)

##### Subtheme 1.2.2: Changes in Child

Parents commonly reported an improvement in their child’s behaviour following attending a parenting programme. In particular, parents described a reduction in behaviour they perceived as problematic, that their child was listening more and had an increased respect for rules and boundaries. One parent noted “it really calmed them down and they really started to listen” (Houlding et al. 2012, p. 2290) and another “He also began to understand the rules, that there are not only rules at home but also in other places” (Errázuriz et al. 2016, p. 3445). Parents also reported changes in their child more widely including their social development, improved confidence and educational attainment.

##### Subtheme 1.2.3: Changes in Family

Following changes in the parent and child, parents frequently reported an overall improvement in the quality of family life as an outcome of attending a parenting programme. One parent described:

I think it changed everyone’s quality of life because I think it was extremely important to realize that applying small strategies we greatly improved situations that were previously very stressful. (Errázuriz et al. 2016, p. 3445)

Parents described clearer expectations within the family and working as a team, making family life feel more manageable. Participants in two-parent families noted an improvement in communication with their partner, feeling closer and more supported by their partner allowing them to co-parent more effectively:

We are working together. When I implement a rule at home, I talk to him. We agree things jointly. (Mejia et al. 2015, p. 681)

#### Subtheme 1.3: Post-intervention

This subtheme related to parents’ experiences following attendance at a parenting programme, the challenges they faced and how they sought to maintain positive outcomes they had derived. One author identified the process by which parents experience cumulative advantage or disadvantage following an intervention (Vella et al. 2015). Some parents described a sense of loss following the end of a parenting programme and frequently highlighted the need for ongoing support following the end of a programme. The perseverance required and the challenges they faced in continuing to implement what they had learnt was identified:

At the end of the 9 weeks, I wasn’t ready for it to be over. (Owens et al. 2007, p. 188)

Sometimes you’re so busy, you just forget… You don’t realize until you see the kids acting up and you think, ‘Oh God, I haven’t played with them in ages’ or even really praised them in the last week. (Furlong and McGilloway 2015, p. 690)

The way in which parents utilised what they had learnt from a parenting programme varied significantly, with some describing the continued use of programme resources, whereas for others there was a process of adapting taught material, finding what works and “setting the skills aside” (Holtrop et al. 2014, p. 751) they perceived they no longer needed. Parents described a need to develop self-acceptance in coping with setbacks and seeking support when necessary. For some, maintaining relationships with other parents following a programme or engaging in other sources of support were important:

I’ve learned that if something happens to say, ‘Ok, forget it. Let’s move on’. Rather than dwelling on their bad behaviour and your bad behaviour and beating yourself up, to just move on. (Furlong and McGilloway 2015, p. 692)

Things hadn’t been going well for a couple of months and I was at a loss. So I contacted them [the service providers] and I was back on track after a couple of weeks. (Furlong and McGilloway 2015, p. 692)

### Theme 2: Aspects Perceived to be Important or Valuable

#### Subtheme 2.1: Group Leader or Facilitator

Parents identified characteristics of those facilitating the parenting programme they perceived to be of value: the group leader or facilitator of the parenting programme demonstrating a supportive and non-judgemental approach were the most frequently cited. One parent noted, “but they [staff] have no accusing fingers…they give you confidence” (Russell et al. 2007, p. 108). Parents valued the facilitators’ ability to instil hope, modelling the techniques being taught, being able to manage dynamics within the groups, facilitate relationships between parents and balancing flexibility, in allowing parents to influence content, whilst maintaining focus on the aims of the programme.

[Staff] give you the confidence to know that what you are doing is okay, that it is the right way, that it is your way and not someone else’s way…they give people hope. (Russell et al. 2007, p. 109)

#### Subtheme 2.2: Programme Content and Delivery

This subtheme captures the aspects of the program content and delivery that parents identified as important. The most frequently cited content that parents perceived to be valuable was recognising the importance of positive attention, including providing praise and rewards to their children. Parents valued a collaborative, non-directive approach to delivery whereby strategies were suggested rather than taught and tailored to meet the specific needs of parents attending the programme. The importance of holding realistic expectations for change was also emphasised, as noted by one parent:

I think it helps if you have realistic expectations I mean, if you want to transform your child into a saint almost, who will listen to you always […] I mean, I don’t intend to change my kids in everything, but to improve some things, and those things are improving. (Errázuriz et al. 2016, p. 3445)

Some parents perceived role play as beneficial in allowing parents an opportunity to practice skills, commit strategies to memory and increase empathy with their child:

Role play was very helpful because…at home with [son] I don’t necessarily have time to replay all of that […] in my head…. I had role played it, so that made it easier to know what I was gonna do. (Holtrop et al. 2014, p. 753)

…and there was that role model of how you felt then when your mum is completely ignoring you, or when your mum actually turns round and stops and listens to you. And that was quite dramatic actually, and I try to actually listen a bit more, it happened yesterday. (Barlow and Stewart-Brown 2001, p. 126)

Parents particularly valued those delivering the programme visiting them at home, providing an opportunity for individualised support:

And having them come to your house was fantastic because they could see the way I was doing things. It really made the program. (Coates et al. 2017, p. 109)

There were additional practical aspects of programme delivery that were perceived as particularly important including the provision of child-care to support parents to be able to attend the programme, the availability of refreshments and ensuring a convenient time and location.

#### Subtheme 2.3: Value of the Group

The group was commonly identified as being of particular value and importance to parents, helping them to feel less alone, providing a sense of belonging and camaraderie, a source of support and an opportunity to build relationships with other parents:

And it feels good to know that you’re not alone. Even when you’re doing your best and you feel like giving up, you’re not alone. (Garcia et al. 2018, p. 291)

Parents highlighted the value of sharing experiences with other parents, providing an ‘outlet’, feeling reassured, normalising the difficulties they were experiencing, allowing a realisation *that “I am not a bad parent”* (Wolfe and Haddy 2001, p. 81) and an opportunity to learn from other parents:

You felt like you were there for each other, and you talked about what you tried and what they tried… we probably learned more from each other than either of us did from the teacher. (Owens et al. 2007, p. 186)

### Theme 3: Challenges or Difficulties

#### Subtheme 3.1: Barriers to Engagement or Attendance

Parents identified a variety of barriers they experienced to engaging in or attending a parenting programme.

##### Subtheme 3.1.1: Fear of Judgement and Distrust of Others

A frequently cited barrier was parents fearing judgement from professionals and other parents or concerns about ‘being told how to parent’:

It’s like, ‘don’t do that’ and ‘don’t do this.’ No one says what can you do and how it worked for them. (Wolfe and Haddy 2001, p. 85)

Parents expressed concerns regarding privacy, distrust or fear of being reported to child protection agencies. Other parents experienced difficulties with the group environment, feeling pressurised to take part in discussions, unable to ask questions or finding it difficult to build relationships.

##### Subtheme 3.1.2: Lack of Support

Another frequently cited barrier was lack of support, particularly from a partner or extended family, making it difficult to attend or implement parenting strategies. This was of particular importance in two-parent families, where only one parent was engaging with a parenting programme. Parents noted that conflict arose in attempting to implement strategies, describing their partner being reluctant to change their parenting approach, serving to highlight divisions or being dismissive of their new learning. However, some described that this was resolved when the non-attending partner was able to witness the positive benefits and learn through example.

##### Subtheme 3.1.3 Systemic Challenges

There were a number of contextual barriers that parents faced in attending a parenting programme including managing competing demands (e.g. involvement of multiple services, financial pressures associated with attendance, working commitments) and being unable to find the time. Many parents also highlighted wider systemic challenges of parenting in the face of significant adversity:

I had been legally evicted when we was right in the middle of the program… and me and my kids were, well they were staying with my brother, then with my mom for three weeks and I was sleeping in my car. I just had so much going on… it’s like I got too much on my plate right now for the program. (Lewis et al. 2016, p. 3767)

Moreover, some parents described feeling overwhelmed with the information provided to them:

…one parent believed that there was so much information given to her on strategies to effectively discipline her children that she found it difficult to apply everything she had learned when needed. (Lewis et al. 2016, p. 3767).

In some instances, parents perceived that the severity of their child’s difficulties meant the programme was ‘not a good fit’ or they were seeking an alternative pharmacological intervention.

#### Subtheme 3.2: Programme Content

The use of ‘time out’ strategies was frequently cited as an aspect of a programme that parents disliked. Other aspects of a programme that parents expressed negative views about included the use of technical language, video not being perceived as relatable, homework and role play not enjoyable, difficulties in finding time for play or other home exercises, content not being perceived as developmentally appropriate or a lack of cultural fit. Some parents expressed that programme content was not consistent with expectations. As one parent noted:

I think, at the start, that it comes across as a bit fluffy … I wasn’t sure at the beginning that they would be dealing with more of the nitty–gritty … the time-out and the discipline. The positive thing doesn’t make much sense at first … it seems too ‘happy clappy. (Furlong and McGilloway 2012, p. 623)

#### Subtheme 3.3: Suggestions for Improvement

Parents made a variety of suggestions as to how the parenting programme could be improved. The most commonly cited suggestions were tailoring programme content to meet the specific needs of families, ensuring resources are culturally appropriate and portray the reality for parents attending the programme. Parents also suggested longer programme duration or the provision of additional sessions to cover content in greater detail and account for the time taken to introduce behavioural change:

I think it could be a bit longer, that it could be monitored more in time because I’m really happy but when one stops coming, one starts to lose the training and that more precise control of things begins to dilute, because, there are behaviors in a child that will not change in a couple of months. (Errázuriz et al. 2016, p. 3446)

I really feel that the course was so good that I just feel that we need to follow it up, even just once a month or something… Because although the course is really good… I’ve forgotten some of it… I need some support to continue it… It’s so difficult… because… for years and years you’ve… been… the way I’ve been brought up, and… then in… 8 or 10 weeks… they totally change your way of… doing things, and then after that you’re left to your own devices… It’s so easy to… go backwards. (Patterson et al. 2005, p. 59)

Many parents expressed that their experience of the parenting programme would have been improved if they had been able to attend with their partner.

## Discussion

This systematic literature review of 26 studies was the first comprehensive synthesis of the parental experiences and perceptions of parenting programmes using qualitative studies. The current review sought to consider what parents’ and carers’ experiences of parenting programmes were. The aims of the review have been fully met and our findings resulted in the identification of key themes in relation to a family’s journey through a parenting programme, the aspects across parenting programmes perceived to be important or valuable to parents and the challenges or difficulties parents faced when engaging with these interventions. This metasynthesis has significantly enhanced the very preliminary findings of Kane et al. (2007). Providing novel insights into the interplay between parental experiences and programme content, in order to develop a comprehensive understanding of the important concepts to be considered in the planning and delivery of parenting programmes.

Identified parental outcomes (skill development, developing understanding and relationship with child, improved well-being and view of self) are in line with previous findings emphasising the utility of parenting programmes (Barlow and Coren 2018; Kane et al. 2007). It is irrefutable that parents perceive the outcomes associated with a parenting programme extend further than changes in their own behaviour. Changes in the child and wider family are evident across parental reports. The skills of the group leader or facilitator, important aspects of programme content (e.g. positive parenting strategies) and delivery (e.g. role play, home visiting and a collaborative, non-directive approach) and the value of the group were emphasised. These observations reflect findings of previous syntheses of barriers and facilitators to accessing parenting programmes (Mytton et al. 2014). The current review findings regarding parental experiences prior and post-intervention and the challenges and difficulties parents face have important implications for improving the acceptability, feasibility and utility of parenting programmes.

### Clinical and Research Implications

By drawing from larger and more diverse samples of parents who experienced a broader range of parenting programmes, aspects pertinent to the successful implementation of evidence-based policy have been highlighted (Law et al. 2009). The present review allows parents, practitioners, commissioners and policy-makers to carefully consider the implications for the provision of parenting programmes.

The current review stresses that parents perceive the skills of practitioners delivering parenting programmes as crucial to their success. Significant training and ongoing opportunities for supervision are likely to be required in ensuring important skills remain central to delivery (Asgary-Eden and Lee 2012; Moore et al. 2015; Shapiro et al. 2012). The features of parenting programmes that parents perceive to be of particular value, including the use of role play and home visits, corroborate the findings of previous literature (Holtrop et al. 2014; Mytton et al. 2014). These features should be important considerations for practitioners and policy-makers and adequate funding should ensure these valued activities are facilitated.

The importance of the group process as a source of support in tackling parental isolation is consistently reported across the studies included in the present review and elsewhere (Levac et al. 2008; Mytton et al. 2014). The learning that parents take from each other may be a key mechanism of change (Barlow and Stewart-Brown 2001; Borden et al. 2010; Levac et al. 2008; Owens et al. 2007). Whilst the benefits of the group process are evident in the current review, the challenges that this presents for some parents have also been highlighted. There is currently no clear evidence regarding whether parent training is more effective delivered in groups or individually (Barlow and Coren 2018). Thus, offering a choice of interventions of varying intensity to suit the needs of families is likely to be beneficial (Sanders et al. 2007).

The current findings suggest that tailoring programme content to meet the individual needs of families is of particular importance to parents. However, this suggestion presents a challenge to practitioners delivering programmes via a structured curriculum or manual to ensure appropriate adaptations are made whilst maintaining programme fidelity. The necessary balance between fidelity and flexibility has been identified (Barrett 2010; Mytton et al. 2014). Delivering parenting programmes across a cross-cultural context also presents real challenges, particularly in light of the ‘deficit narrative’, whereby parenting programmes are presented as a means to ‘fix’ parenting deemed to be inadequate (van Esch and de Haan 2017). Disregarding local ideas and practices are likely to result in a lack of cultural fit; thus, further research should consider mechanisms of change across different cultural settings (Mejia et al. 2015).

In light of concerns regarding the long-term effectiveness of parenting programmes and the maintenance of positive outcomes (Barlow and Coren 2018; Eyberg et al. 1998), the current review highlights the importance of ongoing peer and professional support for parents following the end of a parenting programme in order to maximise and maintain behavioural change. As has previously been emphasised, further research is required to clarify how this can be optimally provided (Eyberg et al. 1998).

The current review clearly demonstrates that the barriers that parents face in attending a parenting programme should not be underestimated. Recruiting and retaining parents to programmes requires a sensitivity to parental context prior to a parenting programme, including the distress and fear of judgement that parents describe. Facilitating this engagement process and taking time to explore and address parental concerns have previously been demonstrated as important to parents for them to commence, participate and complete parenting programmes (Miller and Prinz 2003). Practitioners should be alert to the obligation to participate and the tendency to acquiesce, as reported by parents. This obligation and tendency to acquiesce has important implications for the process by which parents are invited to attend parenting programmes. Court-mandated attendance at such programmes may have the potential to limit the parents’ willingness to engage, although it is clear that many parents make a transition to an intrinsic willingness to participate and are able to derive benefit (Braver et al. 2016; Fackrell et al. 2011; Pollet and Lombreglia 2008). Thus, parental engagement can be considered in terms of change models with recognised utility (Prochaska and DiClemente 1983; Prochaska et al. 2008).

Policy-makers should account for the wider systemic influences and significant adversity that parents face in attempting to fulfil their parenting role (Mytton et al. 2014). Parents’ widely expressed desire to “be a better parent” (Hartwig et al. 2017, p. 506) should be acknowledged by professionals to support the maintenance of a non-judgemental approach, whereby parents’ commitment to doing the best they can, in often tremendously difficult circumstances, is continually recognised (Allen 2011). The transition to parenthood can be particularly challenging for individuals with a history of maltreatment and the complexity of attempting to break intergenerational cycles is highlighted (Christie et al. 2017; Madden et al. 2015). The value of supporting parents in the often difficult process of reflecting on their own experience of being parented is emphasised; the potential benefits of creating the opportunity for this within the delivery of parenting programmes should be considered (Levac et al. 2008; Wolfe and Haddy 2001).

Moreover, the current review emphasises the importance of wider familial support in attempting to implement change. Mockford and Barlow (2004) describe the “unintended consequences” (p. 1) of parental conflict which can arise following attendance at a parenting programme. The involvement of both parents in a programme is perceived by many parents as potentially beneficial (Furlong and McGilloway 2012; Mejia et al. 2016; Stewart-Brown et al. 2004); thus, finding ways of supporting multiple caregivers to attend should be a focus of further work in this area, raising important considerations for policy-makers in promoting parental equality (Castro-García and Pazos-Moran 2016; Parken 2018).

### Strengths and Limitations

Given that the search was limited to studies written in the English language and those published in peer-reviewed journals, publication and language biases need to be acknowledged. Whilst the majority of included studies were conducted in the United States and United Kingdom (*n *= 18), studies from a variety of countries and cultures were identified and included. Despite attempts to ensure trustworthiness and credibility throughout the review process, it is possible that included studies may be influenced by selection bias. It is important to note that only studies of parents who attended or had been invited to attend a parenting programme were included. Some of the included studies involved parents who ‘dropped out’ or failed to attend after agreeing to participate (Duppong-Hurley et al. 2016; Patterson et al. 2005); however, despite these studies the views of these parents and those who decline to participate may be under-represented in the current review. Thus, the understanding of the barriers faced and the reasons parents may not be invited to participate in parenting programmes require further exploration. Moreover, the parents included in the current review could be considered to be a relatively heterogenous group, representing a number of identified sub-groups (e.g. parents experiencing mental health difficulties, homelessness, parents involved in child-welfare agencies, lone parents and low-income parents). It is acknowledged that parenting in these contexts is likely to come with unique challenges which may not be captured in the current review. Despite these limitations, the number and quality of included studies, the breadth of parenting programmes and the variety of parents included in the current review are a clear strength and it is important to note that none of the identified themes were refuted in any of the included studies. Further work in this area should consider the unique experiences of different groups and seek to identify any notable differences.

It is acknowledged that the themes derived are influenced by the judgement and insights of the reviewers. The enterprise of synthesising qualitative research has been contested (Sandelowski and Barroso 2007) and it is acknowledged that the variety of methodologies applied in the studies included in the current review is likely to have influenced the themes that emerged. There is a suggestion that overly large sample sizes may impede the depth of analysis in a metasynthesis (Sandelowski et al. 1997). However, the use of NVivo software allowed for the handling of large amounts of data and facilitated the analytic process. Furthermore, independent review at stages of study selection, quality assessment and identifying themes was included to enhance the trustworthiness and credibility of findings (Tong et al. 2012).

## Conclusion

This the largest and most comprehensive review of the qualitative literature of parents’ perceptions and experiences of parenting programmes to date. The family’s journey associated with attendance at a parenting programme and the potential utility of programmes as a means of early intervention are emphasised. Important considerations for policy development and service delivery are highlighted, in line with the aspects of parenting programmes deemed to be valuable and the challenges and difficulties parents face in attending. Key recommendations for services in the planning and delivery of parenting programmes include ensuring high-quality training and supervision of practitioners, balancing flexibility and fidelity to allow for tailored content to meet individual needs, a sensitivity to parental adversity, the need for wider familial support and the availability of ongoing support following the end of a programme.

## Appendix

See Tables 4 and 5.

**Table 4:** Enhancing transparency in reporting the synthesis of qualitative research (ENTREQ) statement (Tong et al. 2012)

No	Item	Guide and description	Present
1	Aim	State the research question the synthesis addresses	✓
2	Synthesis methodology	Identify the synthesis methodology or theoretical framework which underpins the synthesis and describe the rationale for choice of methodology (e.g. meta-ethnography, thematic synthesis, critical interpretive synthesis, grounded theory synthesis, realist synthesis, meta-aggregation, meta-study, framework synthesis)	✓
3	Approach to searching	Indicate whether the search was pre-planned (comprehensive search strategies to seek all available studies) or iterative (to seek all available concepts until they theoretical saturation is achieved)	✓
4	Inclusion criteria	Specify the inclusion/exclusion criteria (e.g. in terms of population, language, year limits, type of publication, study type)	✓
5	Data sources	Describe the information sources used (e.g. electronic databases (MEDLINE, EMBASE, CINAHL, psycINFO, Econlit), grey literature databases (digital thesis, policy reports), relevant organisational websites, experts, information specialists, generic web searches (Google Scholar) hand searching, reference lists) and when the searches conducted; provide the rationale for using the data sources	✓
6	Electronic Search	Describe the literature search (e.g. provide electronic search strategies with population terms, clinical or health topic terms, experiential or social phenomena related terms, filters for qualitative research and search limits)	✓
7	Study screening methods	Describe the process of study screening and sifting (e.g. title, abstract and full text review, number of independent reviewers)	✓
8	Study characteristics	Present the characteristics of the included studies (e.g. year of publication, country, population, number of participants, data collection, methodology, analysis, research questions)	✓
9	Study selection results	Identify the number of studies screened and provide reasons for study exclusion (e.g. for comprehensive searching, provide numbers of studies screened and reasons for exclusion indicated in a figure/flowchart; for iterative searching describe reasons for study exclusion and inclusion based on modifications to the research question and/or contribution to theory development)	✓
10	Rationale for appraisal	Describe the rationale and approach used to appraise the included studies or selected findings (e.g. assessment of conduct (validity and robustness), assessment of reporting (transparency), assessment of content and utility of the findings)	✓
11	Appraisal items	State the tools, frameworks and criteria used to appraise the studies or selected findings (e.g. Existing tools: CASP, QARI, COREQ, Mays and Pope; reviewer developed tools; describe the domains assessed: research team, study design, data analysis and interpretations, reporting)	✓
12	Appraisal process	Indicate whether the appraisal was conducted independently by more than one reviewer and if consensus was required	✓
13	Appraisal results	Present results of the quality assessment and indicate which articles, if any, were weighted/excluded based on the assessment and give the rationale	✓
14	Data extraction	Indicate which sections of the primary studies were analysed and how were the data extracted from the primary studies? (e.g. all text under the headings “results/conclusions” were extracted electronically and entered into a computer software)	✓
15	Software	State the computer software used, if any	✓
16	Number of reviewers	Identify who was involved in coding and analysis	✓
17	Coding	Describe the process for coding of data (e.g. line-by-line coding to search for concepts)	✓
18	Study comparison	Describe how were comparisons made within and across studies (e.g. subsequent studies were coded into pre-existing concepts, and new concepts were created when deemed necessary)	✓
19	Derivation of themes	Explain whether the process of deriving the themes or constructs was inductive or deductive	✓
20	Quotations	Provide quotations from the primary studies to illustrate themes/constructs and identify whether the quotations were participant quotations of the author’s interpretation	✓
21	Synthesis output	Present rich, compelling and useful results that go beyond a summary of the primary studies (e.g. new interpretation, models of evidence, conceptual models, analytical framework, development of a new theory or construct)	✓

**Table 5:** Matrix of included studies and identified themes

Study		Theme 1: A family’s journey					Theme 2: Aspects perceived to be important or valuable			Theme 3: Challenges or difficulties		
		Prior to parenting programme	Outcomes			Post-intervention	Group leader or facilitator	Programme content & delivery	Value of the group	Barriers to engagement or attendance	Programme content	Suggestions for improvement
			Changes in parent	Changes in child	Changes in family							
1	Wilson et al. (2018)	✓	✓	–	✓	–	✓	–	–	✓	–	–
2	Garcia et al. (2018)	✓	✓	✓	✓	–	✓	✓	✓	✓	✓	–
3	Haskett et al. (2018)	–	✓	–	–	–	–	✓	–	✓	✓	✓
4	Coates et al. (2017)	–	✓	✓	✓	–	–	✓	✓	–	–	✓
5	Hartwig et al. (2017)	✓	✓	✓	–	–	–	–	–	✓	✓	–
6	Errazuriz et al. (2016)	✓	✓	✓	✓	✓	–	✓	–	✓	✓	✓
7	Duppong-Hurley et al. (2016)	✓	–	–	–	–	–	✓	–	✓	–	✓
8	Lewis et al. (2016)	–	✓	–	–	✓	–	✓	–	✓	✓	✓
9	Mejia et al. (2016)	✓	✓	–	–	–	–	✓	✓	✓	✓	✓
10	Vella et al. (2015)	✓	✓	–	–	✓	–	✓	✓	✓	✓	–
11	Furlong and McGilloway (2015)	–	✓	–	✓	✓	–	–	✓	✓	✓	–
12	Mejia et al. (2015)	–	✓	✓	✓	–	–	✓	–	✓	–	–
13	Butcher and Gersch (2014)	✓	✓	–	–	✓	✓	–	✓	✓	–	–
14	Holtrop et al. (2014)	–	✓	–	✓	✓	✓	✓	–	✓	✓	✓
15	Estefan et al. (2013)	–	✓	–	✓	–	✓	–	–	✓	–	–
16	Cullen et al. (2013)	✓	✓	–	✓	–	–	–	–	✓	–	–
17	Houlding et al. (2012)	–	✓	✓	–	✓	–	✓	✓	–	✓	–
18	Furlong and McGilloway (2012)	–	✓	✓	✓	✓		✓	✓	✓	✓	✓
19	Bermudez et al. (2011)	✓	✓	✓	✓	✓	✓	✓	✓	✓	✓	✓
20	Owens et al. (2007)	✓	–	–	–	✓	✓	✓	✓	✓	✓	✓
21	Russell et al. (2007)	✓	✓	–	–	–	✓	✓	✓	✓	–	–
22	Patterson et al. (2005)	✓	✓	–	–	✓	✓	✓	✓	✓	✓	✓
23	Mockford and Barlow (2004)	–	✓	✓	✓	–	–	✓	–	✓	✓	✓
24	Stewart-Brown et al. (2004)	–	✓	✓	✓	✓	–	✓	–	✓	✓	✓
25	Wolfe and Haddy (2001)	✓	✓	–	✓	–	✓	✓	✓	✓	–	–
26	Barlow and Stewart-Brown (2001)	✓	✓	–	✓	✓	✓	✓	✓	✓	–	✓
N (%) of studies in which theme was identified		14 (53.85%)	24 (92.31%)	10 (38.46%)	15 (57.69%)	13 (50.0%)	11 (42.31%)	20 (76.92%)	14 (53.85%)	24 (92.31%)	16 (61.54%)	14 (53.85%)

Key: ✓  Theme present, –  Theme not present
